# Distinct circulating cytokine levels in patients with angiography-proven coronary artery disease compared to disease-free controls

**DOI:** 10.1016/j.ijcrp.2024.200307

**Published:** 2024-07-04

**Authors:** Eveliina Maaniitty, Sami Sinisilta, Juho Jalkanen, Tuija Vasankari, Fausto Biancari, Jarmo Gunn, Sirpa Jalkanen, K.E. Juhani Airaksinen, Maija Hollmén, Tuomas Kiviniemi

**Affiliations:** a Heart Center, Turku University Hospital and University of Turku, POB 52, FI-20521, Turku, Finland; b Vascular Surgery, Turku University Hospital and University of Turku, POB 52, FI-20521, Turku, Finland; c Department of Medicine, South Karelia Central Hospital, University of Helsinki, Valto Käkelän Katu 1, FI-53130, Lappeenranta, Finland; d Medicity Research Laboratory, University of Turku, Tykistökatu 6A, FI-20520, Turku, Finland

**Keywords:** Coronary artery disease, Cytokine, Inflammation, SYNTAX score

## Abstract

**Background:**

Systemic inflammation has a critical role in the development of symptomatic coronary artery disease (CAD). Identification of inflammatory pathways may provide a platform for novel therapeutic approaches. We sought to determine whether there are differences in circulating cytokine profiles between patients with CAD and disease-free controls as well as according to the severity of the disease.

**Methods:**

Case-control study's population consisted of 452 patients who underwent diagnostic invasive coronary angiography due to clinical indications. We measured the serum concentrations of 48 circulating cytokines. Extent of CAD was assessed using the SYNTAX Score in 116 patients. Cytokine differences between groups were tested using Mann-Whitney *U* test and associations with CAD were explored using a logistic regression model.

**Results:**

Overall, 310 patients had angiographically verified CAD whereas 142 had no angiographically-detected coronary atherosclerosis. In multivariable logistic regression models adjusted for age, sex, hypertension, atrial fibrillation, history of smoking and treatment for diabetes and hyperlipidemia, increased levels of interleukin 9 (OR 1.359, 95%CI 1.046–1.766, *p* = 0.022), IL-17 (1.491, 95%CI 1.115–1.994, *p* = 0.007) and tumor necrosis factor alpha (TNF-α) (OR 1.440, 95%CI 1.089–1.904, *p* = 0.011) were independently associated with CAD. Patients with SYNTAX Score>22 had increased levels of stromal cell-derived factor 1 alfa (SDF-1α), beta-nerve growth factor (β-NGF), IL-3 and decreased level of IL-17 compared to those with score ≤22 when adjusted for smoking and use of beta-blockers.

**Conclusions:**

Patients with CAD have distinct circulating cytokine profiles compared to disease-free controls. Distinct cytokines may have pivotal roles at different stages of coronary atherosclerosis. ClinicalTrials.gov Identifier: NCT03444259 (https://clinicaltrials.gov/study/NCT03444259).

## Abbreviations

β-NGF =: Beta-nerve growth factor
CTACK =: Cutaneous T-cell-attracting chemokine
FGFbasic =: Fibroblast growth factor-basic
G-CSF =: Granulocyte colony-stimulating factor
GM-CSF =: Granulocyte-macrophage colony-stimulating factor
GRO-α =: Growth regulated oncogene-alpha
IP10 =: nterferon gamma-induced protein 10
LIF =: Leukemia inhibitory factor
MCP =: Monocyte-chemotactic protein
M-CSF =: Macrophage colony-stimulating factor
MIF =: Macrophage migration inhibitory factor
MIP =: Macrophage inflammatory protein
MIG =: Monokine induced by interferon-gamma
PDGF-BB =: Platelet-derived growth factor-BB
RANTES =: Regulated upon Activation, Normal T Cell Expressed and Presumably Secreted
SCF =: Stem cell factor
SCGF-β =: Stem cell growth factor beta
SDF-1α =: Stromal cell-derived factor 1 alfa
TRAIL =: Tumor necrosis factor-related apoptosis-inducing ligand

## 1 Introduction

Cardiovascular diseases (CVD) are the leading cause of death globally, resulting in estimated of 19 million deaths in 2020 [1]. The number of CVD deaths per year in the United States has been declining from 1980 to 2010 but it has again increased during the past decade [1]. In the European Union, CVD is estimated to cost €282 billion annually to the EU [2]. Modifiable risk factors account for 55 % of CVD endpoints, and according to current understanding, the rest is determined by other factors such as genetics, epigenomics and inflammation [3]. Therefore, there is a need to elucidate the role of inflammatory signaling at different stages of symptomatic coronary artery disease (CAD).

Cytokines are low-molecular weight proteins that modulate inflammation through complex pathways [[4], [5], [6], [7]]. There is emerging evidence that chronic inflammation plays a fundamental role in the development of atherosclerosis and CAD [6]. Cytokines are known to have a role in all stages of atherosclerosis from foam cell and fatty streak formation to plaque rupture and subsequent atherothrombosis [4].

A better understanding of distinct cytokines involved in the development of CAD could provide a novel target for further research and thereby enable targeted drug development. Cytokines could also act as potential biomarkers of CAD. Therefore, the aim of our study was to determine the differences in circulating cytokine profiles in patients having CAD compared to disease-free controls. We also hypothesized, that cytokine profiles could differ according to severity of CAD.

## 2 Methods

The first author has full access to all the data used in the study and takes responsibility for its integrity and the data analysis. The data that support the findings of this study are available from the corresponding author upon reasonable request.

### 2.1 Study population

This study included patients from two prospective studies, the CAREBANK and the FACT studies, that enrolled patients undergoing diagnostic coronary artery angiography for clinical indications at the Heart Center of the Turku University Hospital (Finland). The present study is registered in ClinicalTrials.gov (ClinicalTrials.gov Identifier: NCT03444259). Both studies were approved by the local Ethical Committee of the Hospital District of South-West Finland. Studies conformed with the Declaration of Helsinki as revised in 2002. The patient cohort comprised a total of 469 patients.

CAREBANK is a prospective study of patients undergoing adult cardiac surgery (e.g., coronary artery bypass grafting, heart valve surgery, aortic surgery and resection of cardiac tumors) at the Turku University Hospital since February 2016. Patient records were individually reviewed, and baseline clinical data was collected with a standardized structured data collection protocol by trained personnel [8]. The study has been monitored by third-party licensed data-monitor. Preoperative study blood samples (ethylenediaminetetraacetic acid treated (EDTA) -plasma and serum) were obtained at the same time with routine sampling by the accredited hospital laboratory. Generally, when the operation was elective and patient arrived to the hospital from home, preoperative blood samples were collected one day before surgery, or in the morning before surgery. In patients needing urgent surgery, blood samples were taken before the operation, usually a day before, or on the same day as operation. Database is pseudonymized and data privacy regulation has been followed during the study. Written informed consent for the study was obtained from all patients before the study enrolment. Six patients were excluded from the registry due to missing cytokine measurements or failure in ELISA analyses. The CAREBANK study population consisted of 322 patients.

The FACT cohort was assembled by contacting consecutive patients who had undergone a diagnostic coronary angiography between September 2015 and December 2015 at Turku University Hospital. We contacted a total of 267 patients who fulfilled the eligibility criteria, of them 189 gave written informed consent. EDTA-plasma and serum samples were obtained at certified hospital laboratories. The period between recent major adverse cardiac and cerebrovascular event (MACCE), including non-fatal myocardial infarction, non-fatal stroke and unscheduled coronary revascularization, and blood sampling was required to be ≥ 3 months. A total of 152 patients gave a blood sample and a written consent. Altogether 11 FACT patients were excluded (heart transplant (n = 1), MACCE <3 month (n = 1), cytokine analyses not available (n = 9)). Therefore, the final FACT cohort consisted of 141 patients.

**Fig. 1:**
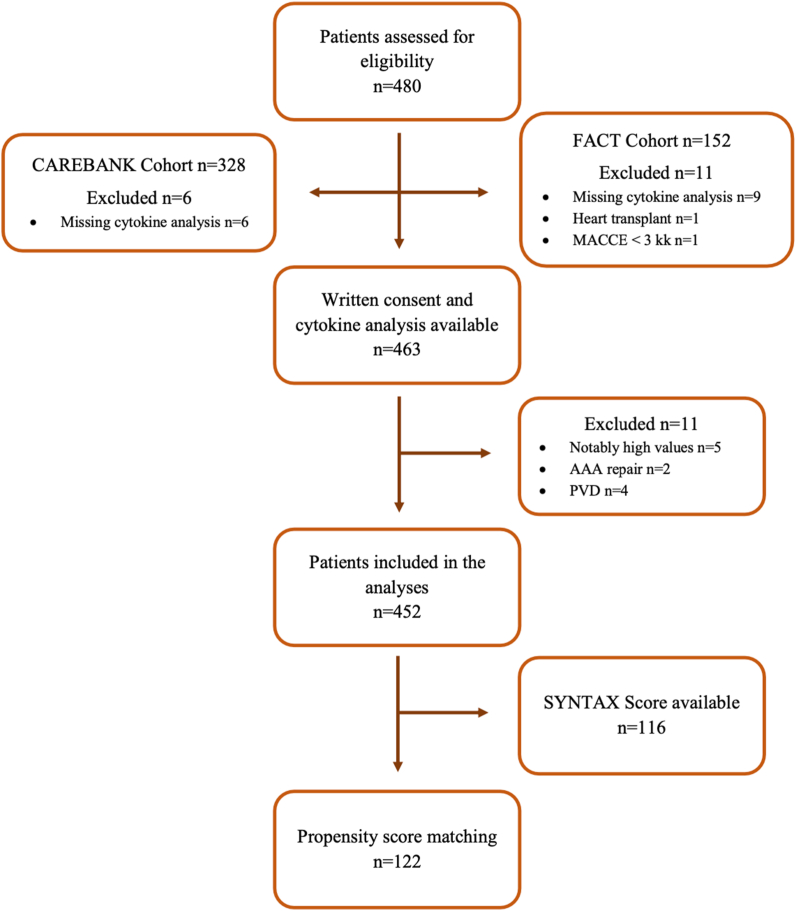
Flow chart of study eligibility and formation of study cohort. *Abbreviations:* MACCE = major adverse cardiac and cerebrovascular event; AAA = abdominal aortic aneurysm; PVD = peripheral vascular disease.

Five patients (four CAD patients and one control) had unexplainable notably high values of various markers and were excluded from the final analyses. Two of these patients had rheumatoid arthritis. We also excluded patients who underwent endovascular stent-grafting for abdominal aortic aneurysm (AAA, n = 2) or peripheral vascular disease (PVD, n = 4) (Fig. 1). The levels of interleukin 2 (IL-2), IL-15, interleukin 12 subunit p40 (IL-12p40) and monocyte-chemotactic protein 3 (MCP-3) were mostly below the detection limit of the assay.

Both study cohorts included patients with stable coronary artery disease and those with acute coronary syndrome. The indications for angiography were stable angina pectoris (n = 127), STEMI (n = 17), NSTEMI/unstable angina pectoris (n = 140), preoperative angiography prior cardiac surgery (n = 135) or diagnostic angiography (n = 30). The indication for angiography was not known in three patients.

The study design is a case-control study. The total patient cohort was divided into two groups depending on whether a significant CAD was detected in coronary angiography. The diagnostic criteria for significant CAD were symptoms related to CAD and >50 % diameter stenosis in one or more coronary arteries. Diabetes, dyslipidemia, hypertension, and pulmonary disease were defined as diseases requiring drug therapy.

### 2.2 SYNTAX Score

We used the SYNTAX Score calculator (available online at syntaxscore.org) to grade the severity of coronary artery disease in the FACT sub-cohort (n = 141). From these patients, 22 did not have CAD and three had notably high values of various markers and were excluded from the final analyses. Final subcohort comprised of 116 patients. Coronary angiographies were evaluated by a trained person in co-operation with an interventional cardiologist. We divided the patients into two groups according to a SYNTAX Score cut-off value of 22 points. The Low SYNTAX Score group (SYNTAX Score ≤22) comprised 86 patients and the High SYNTAX Score (SYNTAX Score >22) comprised 30 patients.

### 2.3 Blood sampling and multiplex analysis

Blood sample analyses were conducted in the MediCity Research Laboratory, University of Turku. We analyzed the levels of 48 different cytokines by using Bio-Plex panel Pro Human Cytokine Screening Panel, 48-Plex (#12007283, BioRad) according to the manufacturer's instructions.

### 2.4 Statistical analysis

Statistical analyses were performed by using IBM SPSS Statistics version 29 and with R 4.3.1 [9] running on macOS 13.5. A *p* value of <0.05 was considered statistically significant. The Shapiro-Wilk -test was used to examine if the cytokine concentration levels fitted into a normal distribution. As the cytokine levels were not normally distributed, a two-sided Mann-Whitney *U* test was used to examine the differences between the case and control groups. The prevalence of cardiovascular risk factors, medications and other baseline characteristics between the groups were compared using the Chi-square test. Variables with a *p* value < 0.05 were entered into a logistic regression model, which was used to explore the associations between clinical variables and cytokine levels. Risk estimates were odds ratios (ORs) with 95 % confidence intervals (CI). The correlation between serum cytokine concentrations and the SYNTAX Score as a continuous variable was explored with the Spearman's rank correlation test.

Propensity score matching (PSM) was used to reduce the effect of differences in baseline characteristics. A propensity score was estimated including the following variables: age, sex, hypertension, atrial fibrillation, history of smoking, type 1 and type 2 diabetes, treatment for diabetes and hyperlipidemia, use of insulin, warfarin, beta-blockers, angiotensin-converting enzyme inhibitors and angiotensin receptor blockers. A 1-to-1 matching was performed using a caliper width of 0.2. All analyses were conducted within the PSM cohort in the same way as described above.

As the analyzes were conducted in two different study cohorts at different times, we used a Z-score to diminish the potential differences due to batch-induced variability in cytokine analyses between the CAREBANK and FACT cohorts. Z-score was calculated for each cytokine separately according to the following equation:

$$Z-score=\frac{x_{i}-\bar{x\left(controls\right)}}{SD}$$

where $x_{i}$ is cytokine concentration of each patient, $\bar{x\left(controls\right)}$ is the mean of cytokine concentrations in healthy controls in each cohort, and SD is standard deviation of cytokine concentration.

## 3 Results

We analyzed the cytokine levels of 452 patients who underwent coronary angiography for clinical indications. Overall, 310 (69 %) patients had angiographically significant CAD (CAD group), whereas 142 had no angiographically significant CAD (control group). The study flow chart is presented in Fig. 1.

### 3.1 Patient cohort

Baseline characteristics are presented in Table 1. CAD patients were older and more often male. Hypertension, dyslipidemia, and diabetes were more prevalent in patients with CAD. In contrast, atrial fibrillation was more prevalent among controls. The proportion of patients with congestive heart failure, obstructive sleep apnea and rheumatic disease were similar between the groups. Use of aspirin, adenosine diphosphate (ADP) receptor inhibitors, calcium channel blockers, beta-blockers, angiotensin converting enzyme inhibitors (ACEi) or angiotensin receptor blockers (ARBs) and insulin were also more common among patients with CAD compared to controls. Propensity score matching yielded 61 pairs with similar baseline characteristics, as expected with the exception of diabetes and antiplatelets drugs.

**Table 1:** Baseline characteristics of total study cohort and propensity score matched cohort of patients with coronary artery disease and disease-free controls.

Baseline characteristics	CAD (n = 310)	Controls (n = 142)	*p* value	PSM CAD (n = 61)	PSM controls (n = 61)	*p* value
Age	68.1 ± 9.6	63.1 ± 12.5	<0.001	68.0 ± 9.9	67.8 ± 9.2	0.931
Female	71 (22.9)	45 (31.7)	0.047	11 (18.0)	18 (29.5)	0.137
Hypertension	260 (83.9)	93 (65.5)	<0.001	53 (86.9)	47 (77.0)	0.158
Atrial fibrillation	65 (21.0)	45 (31.7)	0.014	11 (18.0)	16 (26.2)	0.276
Sleep apnea	38 (12.3)	9 (6.3)	0.056	8 (13.1)	7 (11.5)	0.783
Smoking habit						
Current smoker	40 (12.9)	25 (17.6)	0.191	7 (11.5)	7 (11.5)	1.000
Ex-smoker	140 (45.3)	50 (35.2)	0.044	31 (50.8)	21 (34.4)	0.067
Never smoked	129 (41.7)	67 (47.2)	0.279	23 (37.7)	33 (54.1)	0.069
Diabetes						
Type 1 diabetes	19 (6.2)	0 (0.0)	0.002	2 (3.3)	0 (0.0)	0.154
Type 2 diabetes	91 (29.5)	21 (14.8)	<0.001	25 (41.0)	16 (26.2)	0.085
Heart failure	51 (16.5)	28 (19.7)	0.396	10 (16.4)	8 (13.1)	0.610
Preoperative creatinine (micromol/L)	100.9 ± 64.3	92.0 ± 22.0	0.581	107.8 ± 93.2	97.0 ± 23.5	0.224
Liver cirrhosis	1 (0.3)	0	0.498	0 (0.0)	0 (0.0)	–
Rheumatic disease	29 (8.0)	7 (4.9)	0.107	8 (13.1)	3 (4.9)	0.114
NYHA classes (missing n = 9)			0.017			0.115
I	74 (24.5)	54 (38.0)		15 (24.6)	24 (39.3)	
II	123 (40.7)	52 (36.9)		19 (31.1)	22 (36.1)	
III	91 (30.1)	32 (22.7)		24 (39.3)	14 (23.0)	
IV	14 (4.6)	3 (2.1)		3 (4.9)	1 (1.6)	
CCS classes (missing n = 8)			<0.001			<0.001
I	155 (50.0)	133 (93.7)		32 (52.5)	55 (90.2)	
II	81 (26.7)	4 (2.8)		14 (23.0)	4 (6.6)	
III	56 (18.5)	3 (2.1)		13 (21.3)	2 (3.3)	
IV	11 (3.6)	1 (0.7)		2 (3.3)	0 (0.0)	
**Medications**						
Treatment for dyslipidemia	287 (92.6)	44 (31.0)	<0.001	41 (67.2)	41 (67.2)	1.000
Treatment for diabetes	112 (36.1)	21 (14.8)	<0.001	27 (44.3)	16 (26.2)	0.037
Insulin	58 (12.8)	4 (2.8)	<0.001	15 (24.6)	3 (4.9)	0.002
Warfarin	41 (13.2)	30 (21.1)	0.032	5 (8.2)	11 (18.0)	0.108
DOAC	16 (5.2)	12 (8.5)	0.178	3 (4.9)	5 (8.2)	0.464
ASA	234 (75.5)	28 (19.7)	<0.001	46 (75.4)	20 (32.8)	<0.001
ADP receptor inhibitor	93 (30.0)	2 (1.4)	<0.001	14 (23.0)	1 (1.6)	<0.001
Calcium channel blocker	88 (28.4)	28 (19.7)	0.050	18 (29.5)	14 (23.0)	0.410
Beta-blockers	235 (75.8)	81 (57.0)	<0.001	47 (77.0)	40 (65.6)	0.161
ACEis/ARBs	223 (71.9)	79 (55.6)	<0.001	42 (68.9)	40 (65.6)	0.700

Continuous variables are reported as mean ± standard deviation. Categorical variables are reported as counts and percentages (in parentheses). *Abbreviations: ACEi* = angiotensin-converting enzyme inhibitor; *ADP* = adenosine diphosphate*; ARB* = angiotensin receptor blocker; *ASA* = acetylsalicylic acid; CAD = coronary artery disease; *CCS =* Canadian Cardiovascular Society; *DOAC* = direct oral anticoagulant; *NYHA* = New York Heart Association; *PSM* = propensity score matched.

### 3.2 Cytokine levels

Compared to controls, patients with CAD had increased levels of interleukin (IL) 4, IL-9, IL-17, granulocyte-macrophage colony-stimulating factor (GM-CSF), tumor necrosis factor alpha (TNF-⍺), interferon gamma (IFN-ɣ), platelet-derived growth factor-BB (PDGF-BB) with false discovery rate-adjusted *p* values < 0.050 (Table 2). The levels of IL-5, IL-18 and fibroblast growth factor-basic (FGFbasic) were numerically higher in disease-free controls, but the differences were not statistically significant. Levels of IL-2 and IL-15 were statistically lower in CAD patients, but levels of these cytokines were mostly below the detection limit.

**Table 2:** Cytokines with statistically significant differences between CAD and control groups.

Cytokines	Total Cohort			PSM Cohort		
	CAD	Controls	*p* value	CAD	Controls	*p* value
	Median (IQR)	Median (IQR)		Median (IQR)	Median (IQR)	
IL-17	0.0616	−0.2009	0.001	0.1435	−0.2009	0.034
	(-0.2993–0.6699)	(-0.5045–0.2983)		(-0.2501–0.8569)	(-0.6626–0.2034)	
IL-9	0.3045	−0.0022	0.004	0.4743	−0.0488	0.040
	(-0.3398–1.0498)	(-0.5273–0.5969)		(-0.1986–1.1944)	(-0.7278–0.7134)	
IFN-ɣ	−0.1658	−0.2221	0.017	−0.0831	−0.2587	0.034
	(-0.3469–0.1315)	(-0.4745–0.0447)		(-0.2415–0.1560)	(-0.5077–0.0062)	
TNF-α	−0.0131	−0.1934	0.028	0.1847	−0.3153	0.034
	(-0.3894–0.5006)	(-0.5779–0.4527)		(-0.2733–0.7518)	(-0.6982–0.3829)	
IL-4	−0.1010	−0.2565	0.004	−0.1078	−0.2734	0.114
	(-0.3917–0.5455)	(-0.5094–0.0672)		(-0.3917–0.5591)	(-0.6249–0.1745)	
GM-CSF	−0.3943	−0.4442	0.004	−0.1116	−0.5960	0.114
	(-0.6155–0.7224)	(0.6155–0.1999)		(-0.6155–0.1763)	(-0.6155–0.1763)	
IL-15	−0.1970	−0.1970	0.007	−0.1970	−0.1970	0.072
	(-0.4281–0.1970)	(-0.1970–0.1970)		(-0.4281–0.1970)	(-0.1970–0.1970)	
IL-2	−0.2890	−0.2890 (−0.2890–0.2890)	0.018	−0.2890	−0.2890	0.124
	(-0.4151–0.2890)			(-0.4151–0.2890)	(-0.2890–0.2890)	
PDGF-BB	0.0739	−0.1758	0.028	0.2203	−0.2057	0.226
	(-0.5141–0.5939)	(-0.6930–0.3082)		(-0.4228–0.7490)	(-0.6965–0.5767)	

The Z-scores with 25^th^-75^th^ interquartile range (IQR) of cytokines that achieved a false discovery rate adjusted *p* value <0.05 in the Mann-Whitney *U* test in the total cohort and in the PSM cohort. Cytokines that did not achieve statistical significance are summarized in Supplemental Table 1.

*Abbreviations: CAD* = coronary artery disease; *PSM* = propensity score matched.

**Fig. 2:**
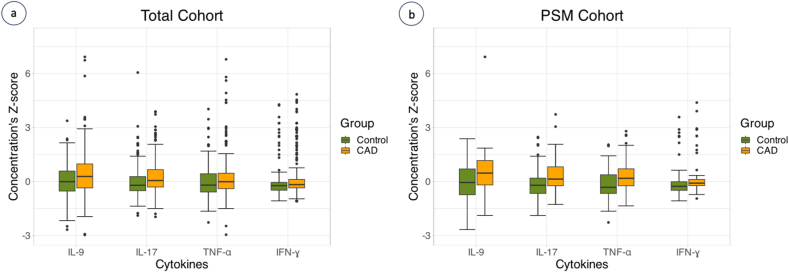
Cytokine levels of IL-9, IL-17, TNF-α and IFN-ɣ. Cytokine concentration's Z-scores in the overall study cohort (a) and in propensity score matched (PSM) cohort (b). Data are shown as medians and 25^th^ and 75^th^ percentiles. The vertical axis represents cytokine concentration's Z-score and horizontal axis different cytokines in the coronary artery (CAD) group (orange) and control group (green). In the total cohort, 13 outliers were excluded from the figure. *Abbreviations:* IFN-ɣ = interferon gamma; IL = interleukin; TNF-α = tumor necrosis factor alpha. (For interpretation of the references to colour in this figure legend, the reader is referred to the Web version of this article.)

When analyses were conducted in the PSM cohort, IL-9, IL-17, IFN-ɣ and TNF-α were still significantly higher among CAD patients (Fig. 2a and b). The results of statistical analyses are summarized in Table 2 and Supplemental Tables S1 and S2.

### 3.3 Predictors of CAD

Risk estimates of all cytokines analyzed are summarized in Supplemental Table S2. Cytokines that achieved a false discovered rate-adjusted *p* value < 0.05 between CAD and control groups, were entered into logistic regression models. In logistic regression models adjusted for age, sex, hypertension, atrial fibrillation, history of previous smoking, diabetes and hyperlipidemia, increased levels of IL-9 (OR 1.359, 95%CI 1.046–1.766, *p* = 0.022), IL-17 (OR 1.491, 95%CI 1.115–1.994, *p* = 0.007) and TNF-α (OR 1.440, 95%CI 1.089–1.904, *p* = 0.011) were independently associated with angiographically detected CAD.

In logistic regression models adjusted for treatment of diabetes conducted in the PSM cohort, IL-9 (OR 1.654, 95%CI 1.129–2.422, *p* = 0.010), IL-17 (OR 1.948, 95%CI 1.259–3.012, *p* = 0.003) and TNF-α (OR 1 2.051, 95%CI 1.286–3.272, *p* = 0.003) were independently associated with CAD.

### 3.4 SYNTAX Score sub-cohort analysis

The baseline characteristics of patients with available SYNTAX Score are summarized in Supplemental Table S3. Rheumatic disease and obstructive sleep apnea were more prevalent in the High SYNTAX Score group, but their frequency was rather low. The history of smoking was more prevalent and the use of beta-blockers more infrequent in the high score patients, otherwise there were no statistically significant differences in baseline characteristics.

Cytokine levels of stromal cell-derived factor 1 alfa (SDF-1α) (*p* = 0.006), β-NGF (*p* = 0.011) and IL-3 (*p* = 0.019) were increased, whereas IL-17 (*p* = 0.040) was decreased in patients with higher SYNTAX Score (Supplemental Fig. S1), and a positive association was detected between β-NGF and SYNTAX Score (*r* = 0.32, *p* = 0.001) and SDF-1α and SYNTAX Score (*r* = 0.30, *p* = 0.001) as shown in the figure (Supplemental Fig. S2).

In multivariable logistic regression models adjusted for history of smoking and use of beta-blockers, elevated level of SDF-1α (OR 2.426, 95%CI 1.214–4.846, *p* = 0.012) and diminished level of IL-17 (OR 0.507, 95%CI 0.259–0.994, *p* = 0.048) were independently associated with higher SYNTAX Score. Further results of the analyses of the SYNTAX Score cohort are summarized in Supplemental Table S4.

### 3.5 Diabetic patients' sub-cohort analysis

We did a sub-cohort analysis including only patients with diabetes. The number of patients with diabetes was higher amongst CAD patients. There were no type 1 diabetics in control group and 19 in CAD group (*p* = 0.002). The number of type 2 diabetics (T2DM) was 91 (30 %) vs 21 (15 %) in CAD and controls groups, respectively (*p* < 0.001). Altogether, there were 131 patients with diabetes in the total cohort, of which 110 had CAD (84 %). Treatment for dyslipidemia was more prevalent in CAD group (96 % vs 67 %) (*p* < 0.001) as was the use of betablockers, aspirin and ADP receptor inhibitors, unexpectedly. The use of warfarin was more common control group (29 % vs 12 %) (*p* = 0.046). Other baseline characteristics did not achieve statistical significance (data not shown).

When cytokine levels were compared between these groups, patients with diabetes and coronary artery disease had elevated levels of IL-6 (*p* = 0.020), IL-9 (*p* = 0.007), IL-10 (*p* = 0.041), IL-17 (*p* = 0.008), FGFbasic (*p* = 0.030), granulocyte colony-stimulating factor (G-SCF) (*p* = 0.010) and PDGF-BB (*p* = 0.021) and decreased levels of IL-1β (*p* = 0.020), IL-4 (*p* = 0.005) and IL-5 (*p* = 0.039) compared to those diabetics with no angiographically significant CAD. When false discovery rate-adjustment was made, no cytokine achieved a significant *p* value. The results of statistical analyses are summarized in Supplemental Table S5.

In a multivariate logistic regression models adjusted with treatment for dyslipidemia and use of warfarin, elevated levels of IL-9 (OR 1.828, 95%CI 1.070–3.157, *p* = 0.027), IL-17 (OR 2.361, 95%CI 1.164–4.789, *p* = 0.017) and TNF-α (OR 2.020, 95%CI 1.041–3.923, *p* = 0.038) were independent predictors of CAD also in diabetic patients. Elevated levels of FGFbasic (OR 3.612, 95%CI 1.371–9.518, *p* = 0.009) and G-CSF (OR 4.169, 95%CI 1.295–13.422, *p* = 0.017) were also associated with CAD.

## 4 Discussion

The present study shows that patients with CAD have distinct circulating cytokine profiles compared to CAD-free controls. The levels of several cytokines were elevated in the CAD group, but there was none with diminished level. The increased levels of IL-9, IL-17 and TNF-α were associated with presence of CAD and levels of SDF-1α and IL-17 were associated with the severity of CAD. To our knowledge, this is the first study to systematically examine this large number of circulating cytokines in angiographically confirmed CAD.

Our study showed elevated levels of many pro-inflammatory cytokines, such as IL-17 [10], TNF-α [11], IFN-ɣ [5] and GM-CSF [5], but also IL-4, which is considered to be an anti-inflammatory cytokine, along with IL-1 receptor antagonist (IL-1RA), IL-10, IL-11 and IL-13 [5,12]. Elevated plasma levels of IL-4 have been reported in patients with CAD, heart failure, heart fibrosis and cardiomyopathies [13]. On the other hand, recombinant IL-4 improved myocardial tissue repair after myocardial infarction in a mouse model [14]. In our study population, diabetic patients with CAD had decreased levels of IL-4 compared to diabetics without CAD, while in total cohort IL-4 was elevated in CAD patients. Diabetic CAD patients have been reported to have both elevated and decreased levels of IL-4 and one study reported elevated levels in T2DM patients and CAD patients but significantly decreased levels in T2DM-CAD patients [15,16]. Previous studies have reported increased concentrations of IL-3 in CAD and its elevated concentrations predicted restenosis after percutaneous coronary intervention [17]. The present analysis demonstrated an association between increased levels of IL-3 and severe CAD as graded by the SYNTAX Score. IL-17 is a proinflammatory cytokine linked to chronic inflammation, but studies of its role in atherosclerosis have been controversial and it has been suggested to have both pro-atherogenic and anti-atherogenic properties [18,19]. Contrary to our findings, previous studies found no difference in circulating IL-17 between patients with or without CAD nor those between stable CAD and those with acute coronary syndrome [20]. However, the healthy control group of this study consisted of only 18 subjects [20]. In our study, lower level of IL-17 was associated with higher SYNTAX Score. Similar finding was reported in a study by Liu et al. [21], but there are also opposite findings with increased level on IL-17 with the severity of CAD [22]. Further, research is needed to establish the precise role of IL-17 in the development of CAD.

IL-9 has been shown to have a significant role in the formation of atherosclerotic plaque in a murine model. Zhang et al. showed that administration of recombinant IL-9 increased plaque size and macrophage and T cell infiltration in the aorta of ApoE −/− mice, and treatment with neutralizing anti-IL-9 monoclonal antibody led to decreased plaque size [23]. Levels of IL-9 have also been reported to be elevated in patients with acute coronary syndrome [23,24] and in patients with carotid artery stenosis [24]. TNF-α is a proinflammatory cytokine that is associated with many inflammatory diseases, such as rheumatoid arthritis, psoriasis and Crohn's disease, and thus anti-TNF-based therapies have been developed to treat these conditions [11]. TNF-α is also considered to be a pro-atherogenic cytokine [25] and patients with rheumatoid arthritis treated with anti-TNF-therapies have been shown to have reduced risk of cardiovascular events [26]. Few studies have linked levels of TNF-α to severity of CAD [22,27]. The present findings suggest that TNF-α has an impact on coronary atherosclerosis, but it does not correlate with the severity of CAD.

Granulocyte-macrophage colony-stimulating factor (GM-CSF) and interferon gamma (IFN-ɣ) are pro-inflammatory cytokines that possess pro-atherogenic features [25] but GM-CSF and IFN-ɣ are also claimed to have anti-atherogenic properties [28,29]. Increased levels of granulocyte colony-stimulating factor (G-CSF) have been observed in CAD patients and in animal models but its role in atherosclerosis remains unclear [28]. In diabetic patients, elevated level of G-CSF was associated with CAD but equivalent finding was not observed in total cohort. G-CSF promotes tissue remodeling and angiogenesis [30] and thus has been studied in tissue repair after myocardial infarction but results are controversial [28,31]. IFN-ɣ is extensively studied in atherosclerosis as it plays a major role in regulation of the immune system, and it has been shown to have effect different stages of atherogenesis [29,32,33]. IFN-ɣ has been found in human atherosclerotic plaques [34] and it also regulates production of other pro-inflammatory and pro-atherogenic cytokines, such as TNF-α and IL-6 [25,32]. Our study supports previous findings that 10.13039/501100021349CAD patients have higher serum levels of IFN-ɣ [35,36] and highlights its role in 10.13039/501100021349CAD and as a potential target for pharmaceutical developments. GM-CSF has a role in atherogenesis, and it has been studied in promoting collateral formation in patients with extensive CAD with controversial results [37,38]. Animal models have also given divergent results with both decreased and increased atherosclerotic plaque area after administration of GM-CSF [39,40]. There is limited data on circulating GM-CSF levels in CAD patients. Our study showed elevated level of GM-CSF in CAD patients and encourage additional research on this topic.

Chemokines are a subgroup of cytokines with chemoattracting properties, and many of them seem to have a role in atherosclerosis [4]. SDF-1α, also known as chemokine CXCL12, has been widely studied in CAD. SDF-1α has been suggested to be a causal mediator of CAD [41] and elevated levels have been reported to predict adverse cardiovascular events [41,42]. It also affects tissue regeneration after myocardial infarction [43]. Ferdousie et al. observed a positive correlation between SDF-1α levels and the Gensini score, which is a grading system of the severity of CAD [44]. We reported similar findings using the SYNTAX Score, although the correlation was of modest magnitude. There are only a few studies on β-NGF and its role in the development of atherosclerosis. Our study showed elevated levels of β-NGF in CAD patients with high SYNTAX Score, and a modest positive correlation with the SYNTAX Score. The correlation between cytokines mentioned above and SYNTAX Score was modest, due to limited number of patients and therefore, residual confounding may exist.

As inflammation and cytokines are strongly associated with atherosclerosis, cytokine-mediated pathways appear as a promising target for cardiovascular drug development. During recent years, several anti-inflammatory drugs have been studied for targeting inflammation in patients with cardiovascular disease with encouraging results. The CANTOS trial [45], which studied the effect of canakinumab, a monoclonal antibody targeting IL-1β, and studies with low-dose colchicine in CAD patients [46,47] demonstrated a reduced risk of cardiovascular events in the treatment groups, which was independent of lipid levels. On the other hand, low-dose methotrexate did not reduce the risk of cardiovascular events compared to placebo in patients with stable CAD [48]. Further research is therefore needed to evaluate the efficacy and safety aspects of anti-inflammatory therapies in atherosclerosis [26].

Even though the standard modifiable cardiovascular risk factors (SMuRFs: hypertension, diabetes mellitus, hypercholesterolemia, smoking) have been well known for decades, the proportion of ST-elevation myocardial infarction patients without any of these risk factors is increasing [49] and these patients have worse short-term mortality [50]. Therefore, there is an apparent need to discover new biomarkers to identify these patients [51]. Cytokines could provide a novel approach of assessing the patients at increased risk of CAD.

### 4.1 Study limitations

The present study has a few limitations which should be considered when interpreting these results. First, the study population is small considering the number of different cytokines explored in the study. This was considered with false discovered rate-adjustment. Second, the blood samples were collected at different times in the study populations. In the CAREBANK population, blood samples were obtained preoperatively, and thus possibly shortly after a major cardiac event. In the FACT study, the period between recent major adverse cardiovascular event and blood sampling was required to be > 3 months. On the other hand, this reinforces the findings that even after several weeks from coronary event, levels of SDF-1α, β-NGF, IL-3 and IL-17 may differ in patients according to the severity of CAD and could therefore be associated with the pathogenesis of more proximal and extensive coronary atherogenesis. Third, in the CAREBANK cohort, many disease-free patients had a valvular defect, for example aortic stenosis, which may affect the cytokine levels. Fourth, several inflammatory cytokines are linked to cancer growth and spread [52]. The coexistence of any malignancy was not investigated in the FACT study and therefore it was not an exclusion criterion in this study. The use of NSAIDs or other pain medications which could affect cytokine pathways were not collected and therefore this has not been assessed.

Finally, Santalahti et al. discovered in their study of insulin resistance, that the correlations between cytokines and insulin resistance disappeared when body mass index was considered as a confounding factor [53]. As the inflamed fat tissue may produce cytokines, obesity should be taken into account [53]. Obesity and body mass index (BMI) were not registered from the whole study population and thus these variables were not taken into account in analyses.

## 5 Conclusions

In conclusions, the present results suggest that a few cytokines may have a significant role in the development of CAD. In addition, some cytokines correlated with severity of CAD, which have been previously unrecognized. Further research is required to establish whether cytokines could be used as biomarkers in clinical settings to identify those patients at high risk of developing CAD.

## Sources of funding

This study was funded by 10.13039/100008723Finnish Medical Foundation, 10.13039/501100005633Finnish Foundation for Cardiovascular Research, State Research Funding (10.13039/501100009420Hospital District of Southwest Finland). Funding sources had no involvement in study design; in the collection, analysis and interpretation of data; in the writing of the report; in the decision to submit the article for publication.

## CRediT authorship contribution statement

**Eveliina Maaniitty:** Writing – original draft, Visualization, Investigation, Formal analysis, Data curation. **Sami Sinisilta:** Investigation, Data curation. **Juho Jalkanen:** Writing – review & editing, Resources, Conceptualization. **Tuija Vasankari:** Writing – review & editing, Investigation, Data curation. **Fausto Biancari:** Writing – review & editing. **Jarmo Gunn:** Writing – review & editing, Conceptualization. **Sirpa Jalkanen:** Writing – review & editing, Resources, Conceptualization. **K.E. Juhani Airaksinen:** Writing – review & editing, Conceptualization. **Maija Hollmén:** Resources, Conceptualization. **Tuomas Kiviniemi:** Writing – review & editing, Supervision, Project administration, Funding acquisition, Conceptualization.

## Declaration of competing interest

Eveliina Maaniitty reports grants in relation to this study from 10.13039/501100005633Finnish Foundation for Cardiovascular Research and 10.13039/501100019689Finnish Cardiac Society.

Tuomas Kiviniemi reports grants in relation to this study from 10.13039/100008723Finnish Medical Foundation, 10.13039/501100005633Finnish Foundation for Cardiovascular Research, State Research Funding (10.13039/501100009420Hospital District of Southwest Finland); in relation to other studies from EU (EIC Pathfinder [MIRACLE], EJCEL H2020 [Moore4Medical]), Atricure, 10.13039/501100006484Vifor Pharma.

K.E. Juhani Airaksinen has received research grants from 10.13039/501100005633Finnish Foundation for Cardiovascular Research and State Research Funding (10.13039/501100009420Hospital District of Southwest Finland) in relation to other studies, and speaker fees from Bayer, Pfizer, and Boehringer Ingelheim, and has been a member of the advisory boards of Bayer, Pfizer, and Astra-Zeneca.

Sami Sinisilta: none; Juho Jalkanen: none; Tuija Vasankari: none; Fausto Biancari: none; Jarmo Gunn: none; Sirpa Jalkanen: none; Maija Hollmén; none.
