# Supplementary material for: Distinct circulating cytokine levels in patients with angiography-proven coronary artery disease compared to disease-free controls
Source: Int J Cardiol Cardiovasc Risk Prev. 2024 Jul 4;22:200307. doi: 10.1016/j.ijcrp.2024.200307 (PMC11292512; doi:10.1016/j.ijcrp.2024.200307)
Supplement: Multimedia component 1 [file mmc1.docx]

**Supplemental Table S1.** Cytokines that did not achieve *p* value <0.05 in the CAD and healthy control groups.

| **Cytokines** | Total Cohort |  |  | PSM Cohort |  |  |
| --- | --- | --- | --- | --- | --- | --- |
|  | CAD  Median (IQR) | Controls  Median (IQR) | *p* value | CAD  Median (IQR) | Controls  Median (IQR) | *p* value |
| IFNα-2 | 0.0731 (-0.7815–0.6090) | -0.3898 (-0.7815–0.4718) | 0.051 | 0.2014 (-0.6984–0.5939) | -0.3160 (-0.7815–0.7862) | 0.6245 |
| IL-5 | 0.0731 (-0.7815–0.6090) | -0.3898 (-0.7815–0.4718) | 0.051 | -0.1591 (-0.4367–0.3403) | -0.3889 (-0.5362--0.1063) | 0.1507 |
| FGF basic | -0.1591 (-0.4367–0.1878) | -0.3175 (-0.5362--0.0890) | 0.055 | 0.0991 (-0.2611–0.3826) | -0.1097 (-0.6081–0.2197) | 0.0720 |
| IL-18 | 0.0145 (-0.2701–0.2935) | -0.0586 (-0.4266–0.2620) | 0.076 | 0.1634 (-0.6443–0.8954) | -0.0971 (-0.9190–0.6832) | 0.4878 |
| IL-1β | 0.1188 (-0.6411–1.0314) | -0.1534 (-0.7811–0.5571) | 0.080 | -0.0356 (-0.2299–0.3309) | -0.1879 (-0.4797–0.1244) | 0.1238 |
| MIG | -0.0717 (-0.3166–0.2808) | -0.1625 (-0.4092–0.1665) | 0.082 | -0.1254 (-0.4210–0.1348) | -0.2356 (-0.5179–0.3655) | 0.7518 |
| MCP-3 | -0.1501 (-0.3888–0.2092) | -0.2558 (-0.4921–0.0696) | 0.082 | -0.2922 (-0.2922--0.2198) | -0.2922 (-0.2922–0.2054) | 0.0720 |
| IL-6 | -0.2922 (-0.2922--0.1575) | -0.2922 (-0.2922--0.0786) | 0.098 | -0.0669 (-0.3582–0.4486) | -0.2882 (-0.6397–0.3569) | 0.1284 |
| IL-1Ra | -0.1525 (-0.4957–0.3438) | -0.2882 (-0.5591–0.2979) | 0.115 | -0.2886 (-0.3971–0.6025) | -0.2036 (-0.5787–0.5531) | 0.5675 |
| M-CSF | -0.1762 (-0.4691–0.5121) | -0.2528 (-0.6131–0.2112) | 0.120 | -0.0488 (-0.4761–0.6125) | -0.2018 (-0.5366–0.3081) | 0.5675 |
| MIP-1β | -0.1011 (-0.4546–0.4475) | -0.2008 (-0.5442–0.1708) | 0.120 | -0.00328 (-0.3842–0.9125) | -0.1756 (-0.6025–0.5693) | 0.3473 |
| LIF | -0.0347 (-0.5577–0.6572) | -0.1727 (-0.6134–0.5597) | 0.145 | -0.2063 (-0.4088–0.3082) | -0.2959 (-0.6245–0.5594) | 0.5675 |
| IL-3 | -0.2311 (-0.5090–0.3248) | -0.3439 (-0.6419–0.2146) | 0.159 | -0.1122 (-0.4236–0.1618) | -0.1906 (-0.6077–0.3178) | 0.5823 |
| Eotaxin | -0.1369 (-0.4525–0.2052) | -0.2275 (-0.6227–0.1656) | 0.162 | -0.0837 (-0.6688–0.8404) | -0.2054 (-0.6838–0.2336) | 0.5675 |
| GROα | -0.0500 (-0.5674–0.7109) | -0.2054 (-0.7144–0.4714) | 0.201 | 0.1089 (-0.6073–0.5317) | -0.1842 (-0.6396–0.3608) | 0.5566 |
| RANTES | -0.0131 (-0.5076–0.6926) | -0.1710 (-0.7025–0.4132) | 0.201 | -0.1919 (-0.3180--0.0442) | -0.2108 (-0.2959--0.0841) | 0.7714 |
| IL-2Ra | -0.2386 (-0.3602--0.1394) | -0.2135 (-0.3173--0.1394) | 0.252 | 0.0009 (-0.4515–0.5597) | -0.0574 (-0.8444–0.5469) | 0.6245 |
| CTACK | -0.0368 (-0.4811–0.5196) | -0.1372 (-0.6819–0.4320) | 0.252 | -0.0880 (-0.6484–0.5857) | -0.2426 (-0.8211–0.7131) | 0.7709 |
| HGF | 0.0450 (-0.5491–0.7613) | -0.0565 (-0.8195–0.6905) | 0.252 | -0.0282 (-0.2569–0.8098) | -0.1271 (-0.2864–0.0516) | 0.2125 |
| β-NGF | -0.0635 (-0.2774–0.2309) | -0.1138 (-0.2716–0.0112) | 0.252 | -0.0232 (-0.5735–0.3635) | -0.2832 (-0.6294–0.2623) | 0.4878 |
| TRAIL | -0.1558 (-0.6079–0.3652) | -0.2810 (-0.6634–0.2266) | 0.252 | -0.2753 (-0.5545–0.0669) | -0.0674 (-0.5571–0.2156) | 0.5339 |
| IL-13 | -0.2343 (-0.5382–0.0897) | -0.1618 (-0.5185–0.2280) | 0.252 | -0.2145 (-0.4104–0.1134) | -0.3342 (0.5742--0.0960) | 0.2622 |
| SCF | -0.2830 (-0.4775–0.1425) | -0.3224 (-0.4775--0.0823) | 0.291 | 0.0008 (-0.5237–0.2742) | -0.1497 (-0.9305–0.5756) | 0.6245 |
| IL-1α | -0.0098 (-0.3809–0.3917) | -0.0762 (-0.5882–0.3515) | 0.302 | -0.2934 (-0.4317–0.1134) | -0.3746 (-0.4317–0.1139) | 0.8436 |
| IL-10 | -0.2959 (-0.4317–0.1392) | -0.3746 (-0.4317–0.0378) | 0.324 | -0.1432 (-0.1982–0.0033) | -0.1759 (-0.1982--0.0598) | 0.6245 |
| G-CSF | -0.1588 (-0.1982–0.0095) | -0.1759 (-0.1982--0.0819) | 0.339 | -0.0182 (-0.2588–0.2074) | -0.1359 (-0.4108–0.2149) | 0.5339 |
| MIP-1α | -0.0685 (-0.3846–0.2436) | -0.1082 (-0.3857–0.2294) | 0.339 | -0.0759 (-0.1911–0.0531) | -0.1319 (-0.2362--0.0315) | 0.2255 |
| TNF-β | -0.1259 (-0.2614–0.0456) | -0.1279 (-0.2368--0.0247) | 0.360 | -0.1644 (-0.6831–0.5896) | -0.2185 (-0.7296–0.3895) | 0.7653 |
| SCGF-β | -0.1782 (-0.6744–0.4708) | -0.2743 (-0.5953–0.1672) | 0.360 | 0.0410 (-0.7625–0.6446) | -0.2223 (-0.8497–0.2238) | 0.5339 |
| IL-12p70 | -0.1027 (-0.7647–0.5436) | -0.1361 (-0.7409–0.3435) | 0.426 | -0.1591 (-0.2573–0.0257) | -0.1714 (-0.2926–0.0084) | 0.5833 |
| IL-8 | -0.1670 (-0.4375–0.1237) | -0.1869 (-0.5638–0.1925) | 0.678 | -0.1441 (-0.4007–0.2885) | -0.1819 (-0.8619–0.1945) | 0.442 |
| IL-7 | -0.1373 (-0.2696–0.1279) | -0.1521 (-0.2608–0.0003) | 0.681 | -0.3027 (-0.5883–0.4487) | -0.1044 (-0.5412–0.4487) | 0.8436 |
| IL-16 | -0.1986 (-0.6430–0.4901) | -0.1986 (-0.5883–0.4335) | 0.779 | 0.0307 (-0.7441–0.4788) | -0.2371 (-0.7873–0.6106) | 0.6979 |
| IP10 | -0.0864 (-0.7145–0.5292) | -0.1176 (-0.8694–0.6106) | 0.808 | -0.1526 (-0.4763–0.1200) | -0.2770 (-0.5051–0.2899) | 0.6979 |
| MCP-1 | -0.2903 (-0.4801--0.0052) | -0.2922 (-0.5474–0.1170) | 0.925 | -0.1789 (-0.6640–0.3120) | -0.1647 (-0.7385–0.3098) | 0.6979 |
| VEGF | -0.1789 (-0.5715–0.3666) | -0.0532 (-0.6972–0.4563) | 0.925 | -0.2307 (0.6001–0.4000) | -0.2756 (0.8156–0.3013) | 0.5339 |
| IL-12p40 | -0.2072 (-0.6700–0.4853) | -0.2052 (-0.7008–0.4035) | 0.925 | -0.2610 (-0.2610--0.2303) | -0.2610 (-0.2610--0.0224) | 0.3668 |
| SDF-1α | -0.2610 (-0.2610–0.1223) | -0.2610 (-0.2610--0.2610) | 0.925 | 0.0836 (-0.4439–0.4746) | -0.1851 (-0.8033–0.6635) | 0.6449 |
| MIF | -0.0543 (-0.5625–0.4520) | -0.1183 (-0.8004–0.6546) | 0.925 | -0.2682 (-0.5381–0.0370) | -0.2100 (-0.6539–0.4196) | 0.7714 |

Values are presented as median Z-scores and 25^th^-75^th^ interquartile range (IQR). Statistical significance was tested using the Mann-Whitney U-test. *Abbreviations:* *CAD* = coronary artery disease; *PSM* = propensity score matched.

**Supplemental Table S2.** Risk estimates of cytokines entered into logistic regression models.

| Cytokines | Total Cohort |  | PSM Cohort |  |
| --- | --- | --- | --- | --- |
|  | Odds ratio (95 % CI) | *p* value | Odds ratio (95 % CI) | *p* value |
| IL-17 | 1.491 (1.115–1.994) | 0.007 | 1.948 (1.259–3.012) | 0.003 |
| TNF-α | 1.440 (1.089–1.904) | 0.011 | 2.051 (1.286–3.272) | 0.003 |
| IL-9 | 1.359 (1.046–1.766) | 0.022 | 1.654 (1.129–2.422) | 0.010 |
| IL-4 | 1.286 (0.998–1.656) | 0.052 | 1.228 (0.923–1.634) | 0.158 |
| IFN-ɣ | 1.283 (0.991–1.662) | 0.059 | 1.423 (0.993–2.039) | 0.055 |
| GM-CSF | 1.212 (0.970–1.514) | 0.090 | 1.420 (0.990–2.038) | 0.057 |
| PDGF-BB | 1.173 (0.871–1.579) | 0.293 | 1.160 (0.833–1.613) | 0.380 |
| IL-15 | 0.888 (0.705–1.118) | 0.312 | 0.668 (0.316–1.409) | 0.289 |
| IL-2 | 1.026 (0.927–1.136) | 0.616 | 0.691 (0.391–1.221) | 0.203 |

The *p* values and odds ratios with 95% confidence interval (CI) from adjusted binary logistic regression models in the total cohort and the PSM cohort. Cytokines that achieved a false discovered rate-adjusted p-value <0.05 between CAD and control groups, were entered into a logistic regression model.

*Abbreviations:* *PSM* = propensity score matched.

**Supplemental Table S3**. Baseline characteristics of SYNTAX Score cohort.

| Baseline characteristics | Low SYNTAX Score (≤22) (n=86)  (%) | High SYNTAX Score (>22) (n=30)  (%) | *p* value |
| --- | --- | --- | --- |
| Mean age | 68.4 ± 11.33 | 69.4 ± 9.11 | 0.838 |
| Female | 33 (38.4) | 6 (20.0) | 0.067 |
| Hypertension | 66 (76.7) | 24 (80.0) | 0.713 |
| Atrial fibrillation | 21 (24.4) | 8 (26.7) | 0.807 |
| Obstructive sleep apnea | 11 (12.8) | 8 (26.7) | 0.077 |
| Smoking (missing n=1) |  |  |  |
| Current smoker | 9 (10.5) | 1 (3.4) | 0.246 |
| Ex-smoker | 38 (44.2) | 19 (65.5) | 0.047 |
| Never smoked | 39 (45.3) | 9 (31.0) | 0.176 |
| Diabetes |  |  |  |
| Type 1 diabetes | 3 (3.5) | 2 (6.7) | 0.460 |
| Type 2 diabetes | 19 (22.1) | 10 (33.3) | 0.221 |
| Heart failure | 19 (22.1) | 7 (23.3) | 0.888 |
| Preoperative creatinine (micromol/L) | 87.2 ± 20.9 | 96.4 ± 37.4 | 0.276 |
| Rheumatic disease | 10 (11.6) | 7 (23.3) | 0.119 |
| NYHA classes  (missing n=8) |  |  | 0.424 |
| I | 16 (20.0) | 5 (17.9) |  |
| II | 37 (46.3) | 9 (32.1) |  |
| III | 23 (28.7) | 11 (39.3) |  |
| IV | 4 (5.0) | 3 (10.7) |  |
| CCS classes  (missing n=7) |  |  | 0.375 |
| I | 40 (49.4) | 13 (46.4) |  |
| II | 24 (29.6) | 5 (17.9) |  |
| III | 13 (16.0) | 7 (25.0) |  |
| IV | 4 (4.9) | 3 (10.7) |  |
| **Medications** |  |  |  |
| Treatment for dyslipidemia | 83 (96.5) | 28 (93.3) | 0.460 |
| Treatment for diabetes | 22 (25.6) | 12 (40.0) | 0.135 |
| Insulin | 10 (11.6) | 3 (10.0) | 0.808 |
| Warfarin | 17 (19.8) | 5 (16.7) | 0.709 |
| DOAC | 5 (5.8) | 2 (6.7) | 0.866 |
| ASA | 64 (74.4) | 24 (80.0) | 0.538 |
| ADP receptor inhibitor | 58 (67.4) | 18 (60.0) | 0.460 |
| Calcium channel blocker | 14 (16.3) | 8 (26.7) | 0.211 |
| Beta-blockers | 70 (81.4) | 19 (63.3) | 0.044 |
| ACEis/ARBs | 64 (74.4) | 24 (80.0) | 0.538 |

Continuous variables are reported as mean and standard deviation (parentheses). Categorical variables are reported as counts and percentages (parentheses) and continuous variables as mean ± standard deviation.

*Abbreviations:* *ACEi* = angiotensin-converting enzyme inhibitors; *ADP* = adenosine diphosphate; *ARB* = angiotensin receptor blocker; *ASA* = acetylsalicylic acid; CAD = coronary artery disease; *CCS =* Canadian Cardiovascular Society; *DOAC* = direct oral anticoagulant; *NYHA* = New York Heart Association.

**Supplemental Table S4.** Results of the SYNTAX Score cohort analyses.

| Cytokines | Low SYNTAX Score (≤22)  Median (IQR) | High SYNTAX Score (>22)  Median (IQR) | *p* value | Odds Ratio (95 % CI) | *p* value |
| --- | --- | --- | --- | --- | --- |
| SDF-1α | -0.3971 (-0.8251--0.0477) | -0.1002 (-0.4439–0.3625) | 0.006 | 2.426 (1.214–4.846) | 0.012 |
| β-NGF | -0.2210 (-0.4686–0.1923) | 0.0149 (-0.2956–0.4060) | 0.011 | 1.462 (0.886–2.412) | 0.137 |
| IL-3 | -0.2319 (-0.4667–0.1075) | -0.0221 (-0.3103–0.3627) | 0.019 | 1.637 (0.930–2.879) | 0.087 |
| IL-17 | 0.2062 (0.0012–0.7116) | 0.0480 (-0.3729–0.5674) | 0.040 | 0.507 (0.259–0.994) | 0.048 |
| MIG | -0.2179 (-0.3543--0.0681) | -0.1609 (-0.2806–0.3384) | 0.056 | 1.264 (0.620–2.579) | 0.519 |
| IL-12p70 | 0.0617 (-0.5132–0.5952) | -0.3439 (-0.8306–0.3578) | 0.069 | 0.943 (0.783–1.136) | 0.538 |
| CTACK | 0.0424 (-0.5043–0.7769) | 0.6667 (-0.3913–1.5695) | 0.086 | 1.433 (0.988–2.078) | 0.058 |
| IL-10 | -0.1608 (-0.5209–0.3159) | -0.3830 (-0.7356--0.0442) | 0.091 | 0.979 (0.889–1.079) | 0.672 |
| IL-9 | 0.6998 (0.0630–1.3558) | 0.1915 (-0.4456–0.3159) | 0.098 | 0.980 (0.909–1.056) | 0.592 |
| TNF-β | -0.1920 (-0.5989–0.4518) | 0.0493 (-0.4775–0.8242) | 0.131 | 1.352 (0.864–2.114) | 0.187 |
| SCF | -0.1509 (-0.3557–0.2872) | -0.0020 (-0.2364–0.4659) | 0.133 | 1.638 (0.810–3.313) | 0.169 |
| IFNα-2 | -0.0280 (-0.7830–0.4753) | 0.2403 (-0.5902–0.7792) | 0.150 | 1.465 (0.938–2.287) | 0.093 |
| MCP-3 | -0.4801 (-0.4801--0.0704) | -0.4091 (-0.4801–0.6015) | 0.153 | 1.331 (0.932–1.901) | 0.115 |
| LIF | -0.1651 (-0.4815–0.4380) | -0.0112 (-0.3254–0.8471) | 0.163 | 1.392 (0.789–2.454) | 0.253 |
| GROα | -0.0122 (-0.8840–0.8491) | -0.4065 (-0.3830–1.5245) | 0.181 | 1.209 (0.914–1.599) | 0.183 |
| IL-7 | -0.3027 (-0.6430–0.3398) | 0.1049 (-0.6430–0.3398) | 0.207 | 1.175 (0.853–1.617) | 0.323 |
| FGFbasic | -0.1294 (-0.3363–0.2037) | -0.1939 (-0.4133–0.1416) | 0.281 | 0.661 (0.248–1.765) | 0.409 |
| Eotaxin | 0.1064 (-0.6867–0.7325) | 0.3059 (-0.4543–0.9555) | 0.289 | 1.044 (0.731–1.493) | 0.812 |
| IL-12p40 | -0.3486 (-0.3486–0.1603) | -0.3468 (-0.3468–0.4439) | 0.323 | 1.331 (0.939–1.887) | 0.108 |
| HGF | -0.0428 (-0.5052–0.7759) | 0.2747 (-0.2383–1.0925) | 0.325 | 1.019 (0.791–1.313) | 0.885 |
| GM-CSF | -0.3854 (-0.4442–1.8310) | -0.442 (-0.4442–1.0013) | 0.347 | 0.994 (0.871–1.133) | 0.923 |
| IL-2Ra | -0.0954 (-0.4811–0.3971) | -0.1502 (-0.2985–0.6853) | 0.349 | 1.231 (0.770–1.969) | 0.385 |
| IL-2 | -0.4151 (-0.4151--0.4151) | -0.4151 (-0.4151--0.4151) | 0.356 | 0.997 (0.944–1.053) | 0.916 |
| IL-1α | -0.2103 (-0.5383–0.3286) | -0.0559 (-0.5369–0.4978) | 0.362 | 1.162 (0.706–1.912) | 0.556 |
| IL-4 | -0.0052 (-0.3945–0.6357) | 0.1135 (-0.1856–0.7283) | 0.372 | 1.251 (0.732–2.139) | 0.412 |
| TNF-α | -0.0925 (-0.3837–0.3148) | -0.2882 (-0.5121–0.2776) | 0.384 | 1.033 (0.784–1.361) | 0.820 |
| IL-18 | 1.2913 (-0.4300–2.6180) | 1.5858 (0.1608–3.1168) | 0.396 | 1.099 (0.934–1.293) | 0.253 |
| VEGF | -0.1151 (-0.5458–0.4951) | -0.1382 (-0.6803–0.1833) | 0.412 | 0.923 (0.599–1.423) | 0.716 |
| IL-15 | -0.4281 (-0.4281–0.4281) | -0.4281 (-0.4281–0.4281) | 0.430 | 1.330 (0.893–1.980) | 0.160 |
| M-CSF | -0.0895 (-0.4477–0.5165) | 0.0004 (-0.2832–0.7098) | 0.457 | 1.081 (0.678–1.724) | 0.744 |
| IL-13 | -0.2830 (-0.5315–0.1312) | -0.4072 (-0.6741–0.0691) | 0.468 | 0.993 (0.637–1.547) | 0.974 |
| IP10 | -0.2452 (-0.3992--0.0204) | 0.2194 (-0.4161–0.1501) | 0.480 | 0.859 (0.380–1.939) | 0.714 |
| MIP-1β | -0.1120 (-0.6670–0.9811) | -0.1452 (-0.6557–0.3983) | 0.524 | 0.814 (0.543–1.220) | 0.318 |
| MIF | -0.3500 (-0.5584–0.0236) | -0.4133 (-0.5904--0.1648) | 0.543 | 0.783 (0.399–1.538) | 0.478 |
| SCGF-β | 0.5450 (-0.6582–1.6518) | 0.4604 (-0.1999–1.7711) | 0.652 | 1.119 (0.864–1.448) | 0.395 |
| IL-6 | -0.2358 (-0.3899--0.0153) | -0.2602 (-0.4336–0.6463) | 0.733 | 0.985 (0.766–1.266) | 0.904 |
| IL-8 | -0.1593 (-0.2055–0.0619) | -0.1538 (-0.2212--0.1048) | 0.741 | 0.376 (0.014–9.870) | 0.558 |
| MIP-1α | -0.1466 (-0.5202–0.3785) | -0.1051 (-0.6198–0.3660) | 0.776 | 1.002 (0.591–1.699) | 0.994 |
| RANTES | -0.3755 (-0.5192--0.0538) | -0.3897 (-0.5156–0.0914) | 0.781 | 0.789 (0.409–1.522) | 0.480 |
| IL-1β | -0.0998 (-0.3962–0.1045) | -0.1659 (-0.4523–0.3098) | 0.837 | 1.156 (0.694–1.927) | 0.578 |
| PDGF-BB | -0.0787 (-0.4917–0.6019) | 0.0545 (-0.5729–0.7188) | 0.843 | 1.128 (0.708–1.794) | 0.613 |
| IL-1Ra | -0.2991 (-0.5021–0.1758) | -0.2238 (-0.5021–0.4961) | 0.865 | 1.218 (0.933–1.591) | 0.147 |
| MCP-1 | -0.3418 (-0.6517–0.0925) | -0.3418 (-0.7385–0.5906) | 0.872 | 1.260 (0.897–1.769) | 0.182 |
| IL-5 | -0.1263 (-0.4367–0.3403) | -0.1263 (-0.3111–0.3419) | 0.878 | 0.940 (0.608–1.454) | 0.781 |
| G-CSF | -0.2854 (-0.6204–0.0126) | -0.3038 (-0.5743–0.2436) | 0.927 | 1.010 (0.577–1.769) | 0.971 |
| IL-16 | 0.2765 (-0.3221–1.1801) | 0.1622 (-0.4137–1.5817) | 0.935 | 1.006 (0.780–1.298) | 0.963 |
| IFN-ɣ | -0.0947 (-0.2243–0.0894) | -0.1220 (-0.2726–0.2366) | 0.980 | 1.143 (0.621–2.103) | 0.668 |
| TRAIL | -0.3769 (-0.8145–0.2369) | -0.4146 (-0.8688–0.4761) | 0.990 | 1.191 (0.917–1.546) | 0.190 |

The Z-scores with 25^th^-75^th^ interquartile range (IQR) of SYNTAX Score cohort in the Low SYNTAX Score (≤22) and the High SYNTAX Score (>22) groups. Risk estimates are odd ratios with 95% confidence interval (CI).

**Supplemental Table S5.** Cytokines that achieved *p* value <0.05 in the CAD and control groups in diabetic patients.

| **Cytokines** |  |  |  |
| --- | --- | --- | --- |
|  | CAD  Median (IQR) | Controls  Median (IQR) | *p* value |
| IL-1β | -0.039 (-0.300-0.281) | -0.304 (-0.480--0.007) | 0.020 |
| IL-4 | -0.108 (-0.358-0.546) | -0.392 (-0.500-- 0.155) | 0.005 |
| IL-5 | -0.149 (-0.437-0.125) | -0.389 (-0.536--0.293) | 0.039 |
| IL-6 | -0.032 (-0.434-0.588) | -0.288 (-0.627-0.038) | 0.020 |
| IL-9 | 0.266 (-0.181-1.050) | -0.347 (-0.730-0.413) | 0.007 |
| IL-10 | -0.1522 (-0.198-0.010) | -0.198 (-0.198--0.143) | 0.041 |
| IL-17 | 0.074 (-0.271-0.639) | -0.271 (-0.897-0.029) | 0.008 |
| FGFbasic | 0.034 (-0.229-0.344) | -0.093 (-0.613-0.127) | 0.030 |
| G-CSF | -0.045 (-0.276-0.263) | -0.204 (-0.775--0.091) | 0.010 |
| PDGF-BB | 0.122 (-0.496-0.768) | -0.206 (-0.640-0.029) | 0.021 |

Values are presented as median Z-scores and 25^th^-75^th^ interquartile range (IQR). Statistical significance was tested using the Mann-Whitney U-test.

*Abbreviations:* *CAD* = coronary artery disease; FGFbasic= fibroblast growth factor-basic; G-CSF= granulocyte colony-stimulating factor; IL-1β= interleukin-1 beta; IQR=25th-75th interquartile range; PDGF-BB= platelet-derived growth factor -BB.


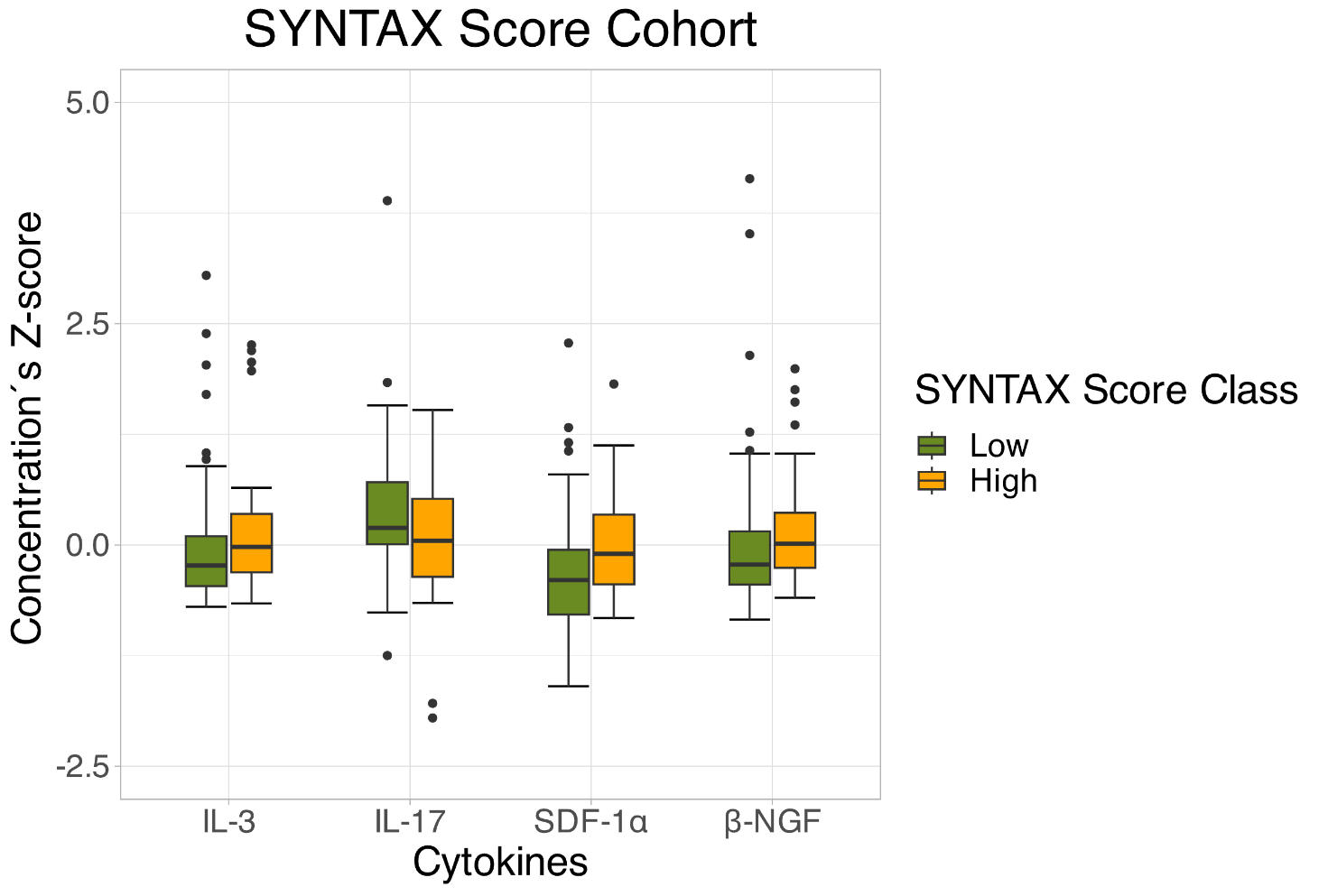


**Supplemental Figure S1. Z-scores of IL-3, IL-17, SDF-1α and β-NGF concentrations in the SYNTAX Score sub-cohort.**

Data are shown as medians and 25th and 75th percentiles. The vertical axis represents cytokine concentration’s Z-score and horizontal axis represents different cytokines in the Low SYNTAX Score group (green) and the High SYNTAX Score group (orange). One outlier was excluded from the figure.

*Abbreviations:* β-NGF = beta-nerve growth factor; IL = interleukin; SDF-1α = stromal cell-derived factor 1 alfa.

b

a


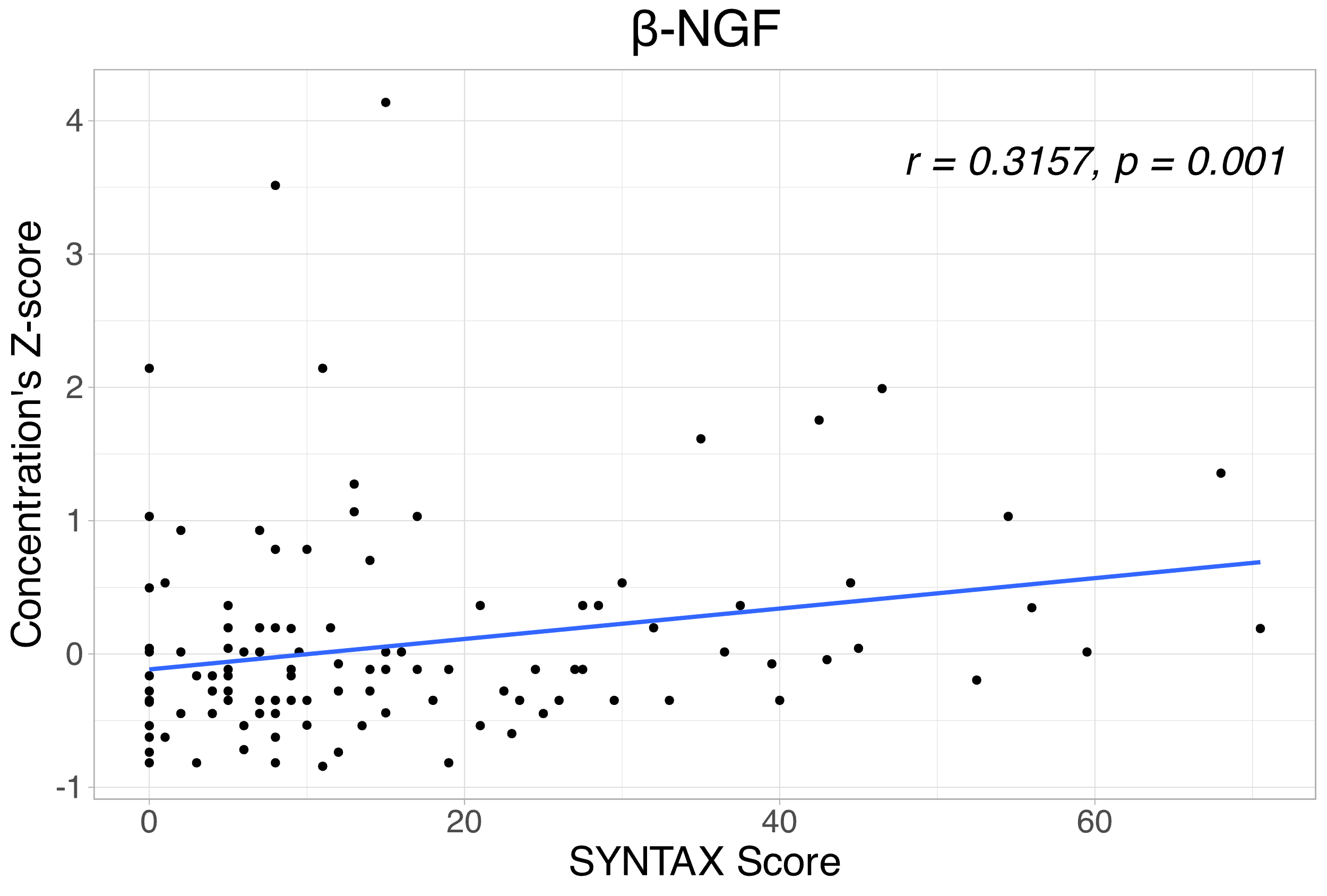

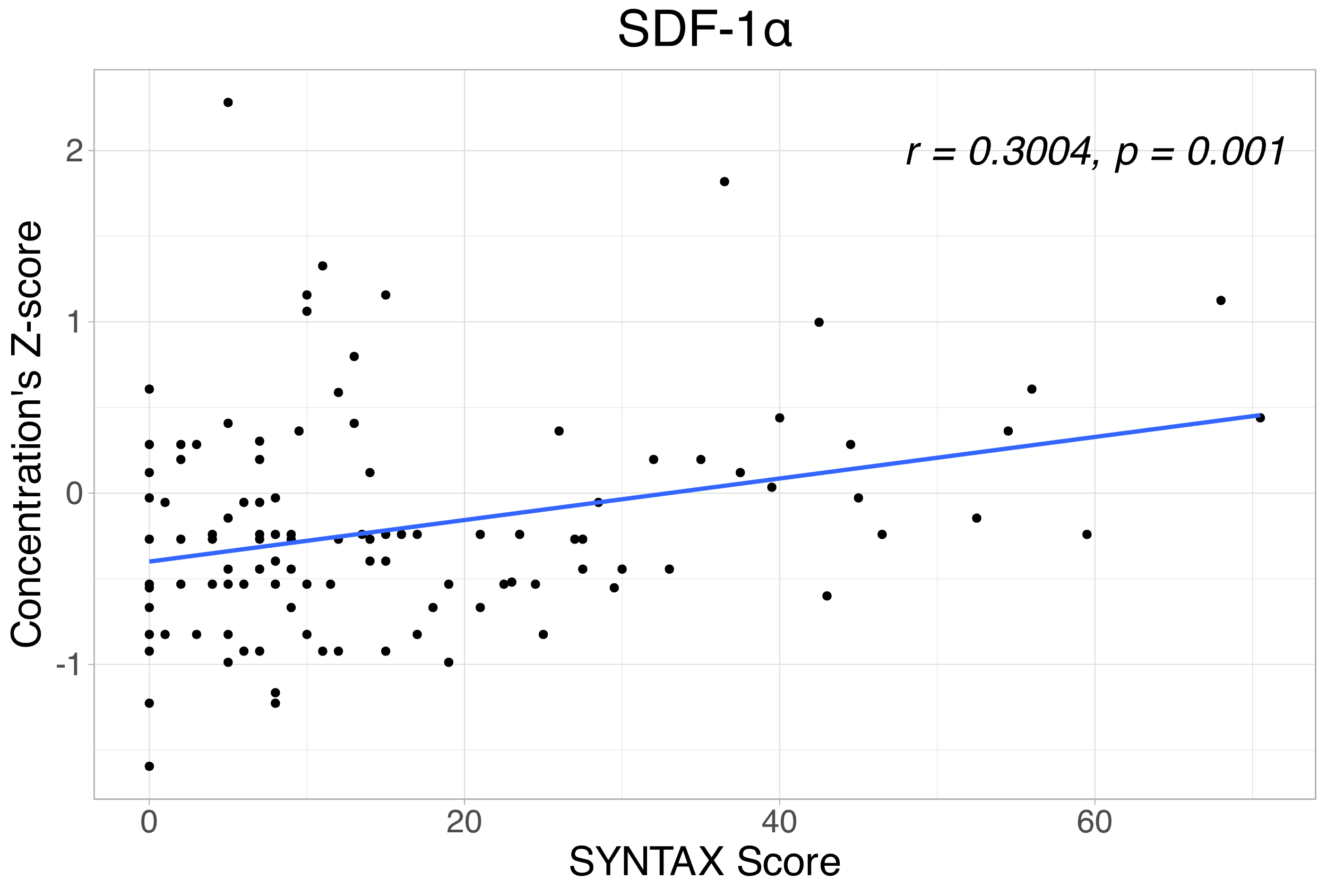

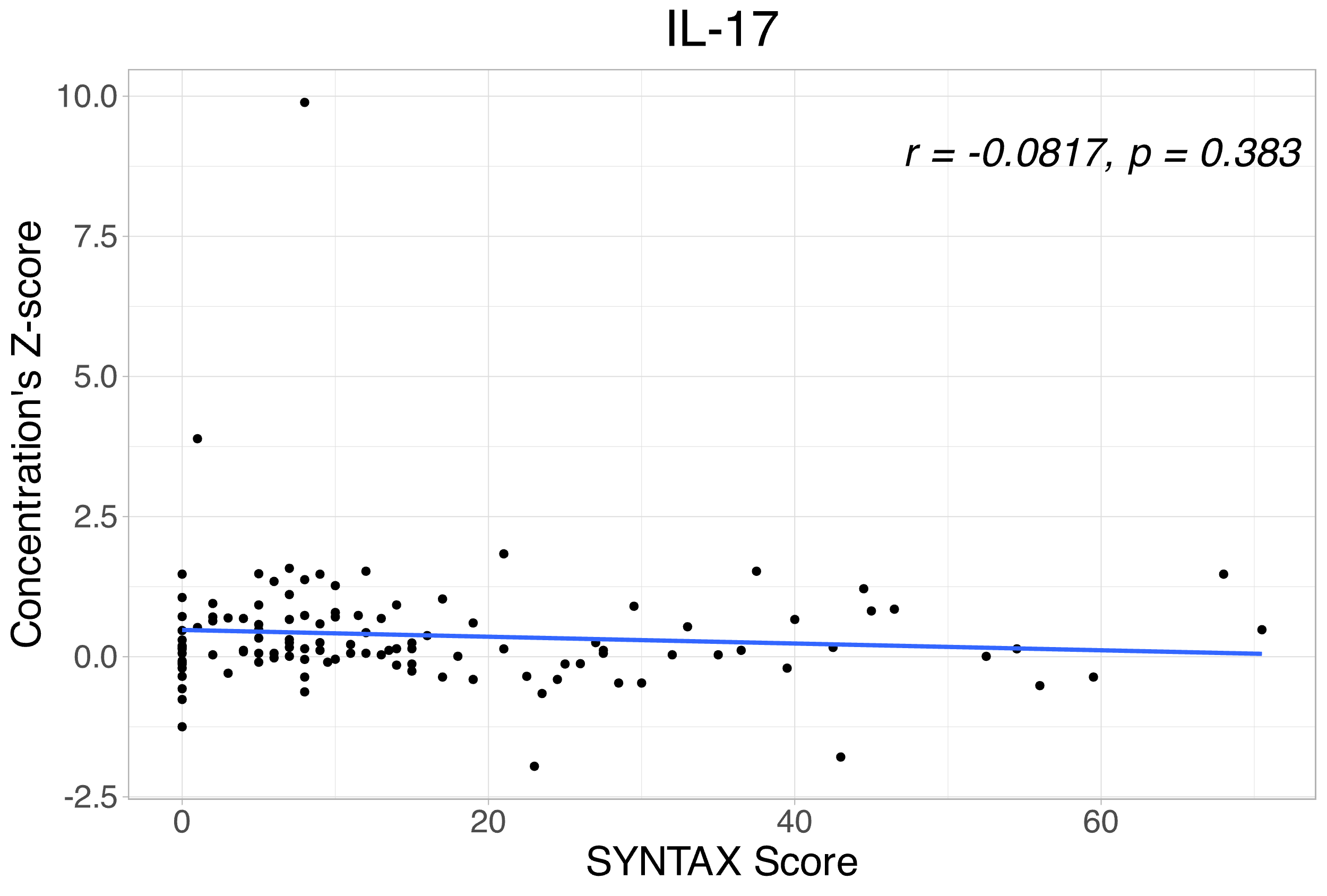

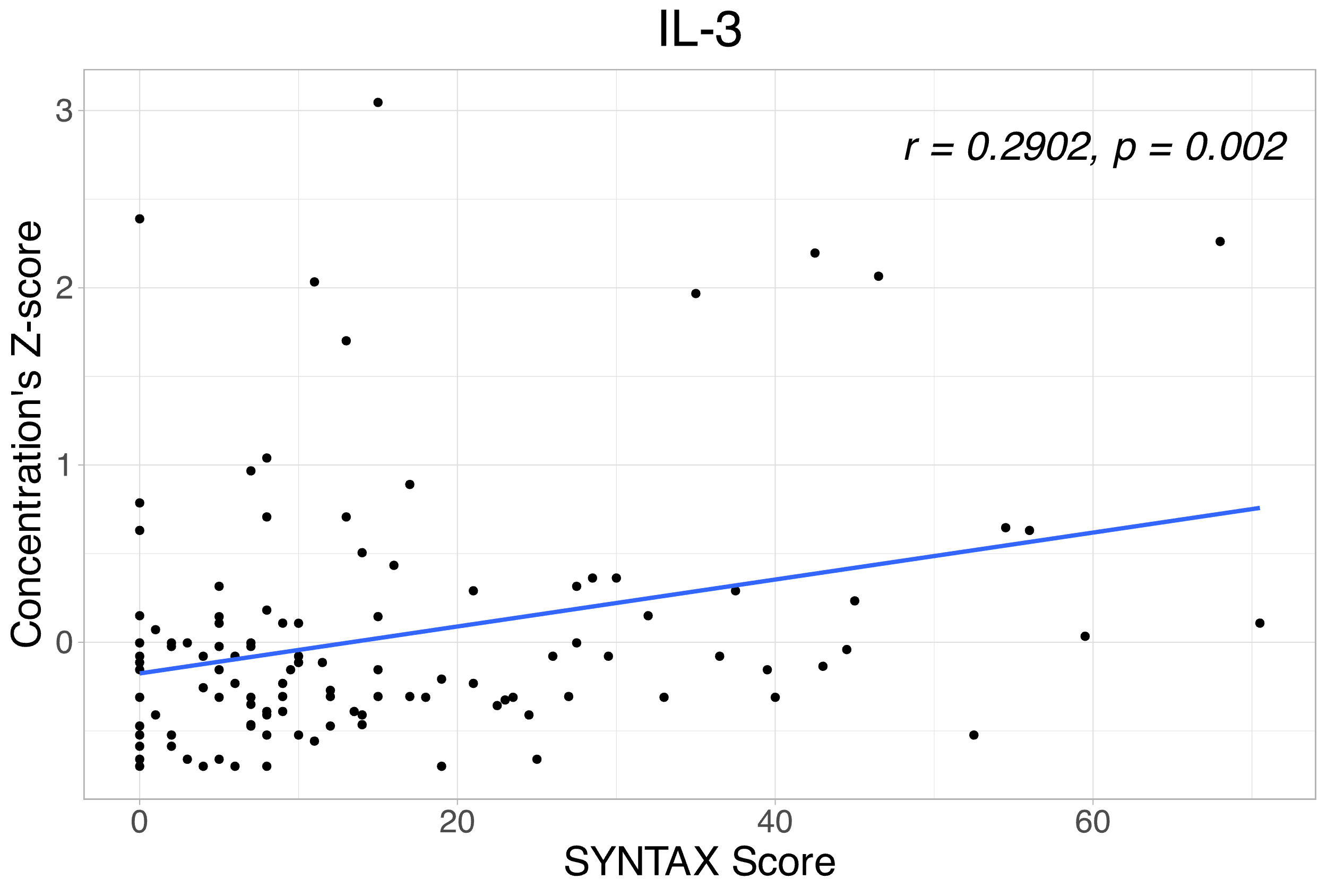


c

d

**Supplemental Figure S2. The correlation of cytokine concentration’s Z-scores with the SYNTAX Score.**

The correlation between cytokine concentration’s Z-score of β-NGF (a), SDF-1α (b), IL-17 (c) and IL-3 (d) with SYNTAX Score. The result of Spearman’s rank correlation test are presented for each correlation. The vertical axis represents cytokine concentration’s Z-score and horizontal axis the SYNTAX Score.

*Abbreviations: β-NGF* = beta-nerve growth factor; *IL* = interleukin; *r* = Spearman’s r coefficient; *SDF-1α =* stromal cell-derived factor 1 alfa.
